# Nostrum Certifiers

**Published:** 1846-06

**Authors:** 


11. *Nostrum Certifiers.*—One of the most successful measures adopted by the nostrum mongers to introduce their vile compounds to public notice, is, securing the names of influential persons to certify that extraordinary cures have been performed by the remedy. Thus we find scores of ministers, lawyers, judges, militia colonels, and, we regret to add, even *physicians*, lending their names and influence to such disreputable purposes. Believing it due to the profession that all of its regular members, who so far forget their own dignity and interests as to become the eulogizers of empirics, should be exposed, we have determined to publish, from time to time the names of all such that come under our notice. It is not improbable that, in some instances, the names of physicians may have been used without their consent, if so, we will take great pleasure in stating the fact when duly notified.

The following persons are reported in *Jayne's Medical Advertiser*, and other papers, as giving certificates in favor of the remedies designated:

M. L. Knapp, M. D., Prof. of Materia Medica, in Laporte University; late Physician to the Baltimore Dispensary, certifies in favor of *Jayne's Carminative.*

R. W. Williams, M. D., Modesttown, Va., Clergyman and Physician, certifies for *Jayne's Expectorant.*

Luther Brigham, M. D., Lowell, Mass., certifies for *Jayne's Expectorant.*

John Quigley, M. D., Sheperdstown, Va., certifies for *Jayne's Hair Tonic.*

S. S. Fitch, M. D., 172 Chestnut Street, Philadelphia, for the same.

Wm. Bacon, M. D., Woodstown, N. Y., certifies for *Jayne's Carminative.*

The following names are given as persons who "*are willing to recommend*" *Beckwith's Anti-Dyspeptic Pills:*

Dr. R. C. Bond, Halifax, N. C.

Dr. Elijah Crosby, Indiana.

Dr. J. Y. Young, Tenn.

Dr. Calvin Jones, Tenn.

Dr. N. L. Smith, Raleigh, N. C.

Dr. J. Manny, Beaufort, N. C.

Dr. T. J. Johnson, Natchez, Miss.

Dr. E. G. Mygatt, Hannibal, N. Y.

Dr. W. R. Scott, Raleigh, N. C.

Dr. N. H. Edwards, Baltimore, certifies for *Spencer's Pills and Bitters.*

Dr. Mattison, Benton co., Ala., certifies for *Hull's Pills.*

Dr. Wm. B. Baker, Springfield, Ky., certifies for *Wistar's Balsam of Wild Cherry.*—*Western Lancet* for May.

